# Consider inequality: Another consequence of the coronavirus epidemic

**DOI:** 10.7189/jogh.10.010359

**Published:** 2020-06

**Authors:** Elisa M Maffioli

**Affiliations:** Department of Health Management and Policy, School of Public Health, University of Michigan, Ann Arbor, Michigan, USA

This perspective piece describes how policymakers should consider inequalities as potential long-term consequences of the current coronavirus epidemic. In addition to health and economic costs, governments and the international community should consider the repercussions of their mitigation and suppression measures, as they might deepen inequalities between the developing and developed world, poor and rich within societies, women and men, and vulnerable populations. This is a call to action for more coordinated efforts at the international level that does not turn a blind eye on this aspect of the pandemic.

The developed world is facing an unprecedented epidemic of a novel pathogen, COVID-19. Worldwide more than 1.4 million confirmed cases and 83 000 deaths have been recorded, as of April 8, 2020 [1]. Currently, developed countries, including the United States of America, Italy, Spain, France and Germany, are the epicenter of this virus. As an increasing number of African and South-East Asian countries have started seeing their first cases, the Global South is preparing for the epidemic to hit.

Governments around the world are acting to reduce the reproduction number R0 (the average number of new infections caused by each infected person) below one and flatten the curve of the contagion. Mitigation and suppression measures include: closing borders, closing schools, case isolation of symptomatic individuals and their contacts, social distancing, and widescale lockdowns of populations [2]. By observing how developed economies are struggling to contain this infectious disease, governments in less developed countries are implementing similar measures. Rwanda was the first country in Africa to impose a lockdown on March 21, 2020, when 11 cases were recorded. Zimbabwe followed with a 21-day lockdown on March 23, 2020, when it only had 2 cases. India issued a 21-day lockdown for the country’s 1.3 billion people on March 24, 2020, as more than 400 cases were recorded. South Africa, Lesotho, and Uganda imposed lockdowns on March 26, March 28, and April 1, 2020, respectively. Sierra Leone even announced a year-long state of emergency despite no confirmed coronavirus. Unfortunately, despite early actions, the coronavirus outbreak is expected to threaten disproportionately the economies of already impoverished countries, both in terms of the disease burden and in terms of social and economic damages.

Countries in the developing world have already experienced and survived other crises, which deteriorated their economies. Among others, West Africa and the DRC were hit by Ebola in 2014-2016 and 2018-2020, respectively. Mozambique was affected by Cyclone Idai in 2019, and billions of desert locusts swarmed Somalia, Ethiopia, and Kenya earlier this year. Conflicts, such as the recent Rohingya crisis in Northern Myanmar, forced the displacement of millions of people. However, nothing seems to be on the scale of what is expected for COVID-19 [3], which is projected to have worse consequences in developing countries.

**Figure:**
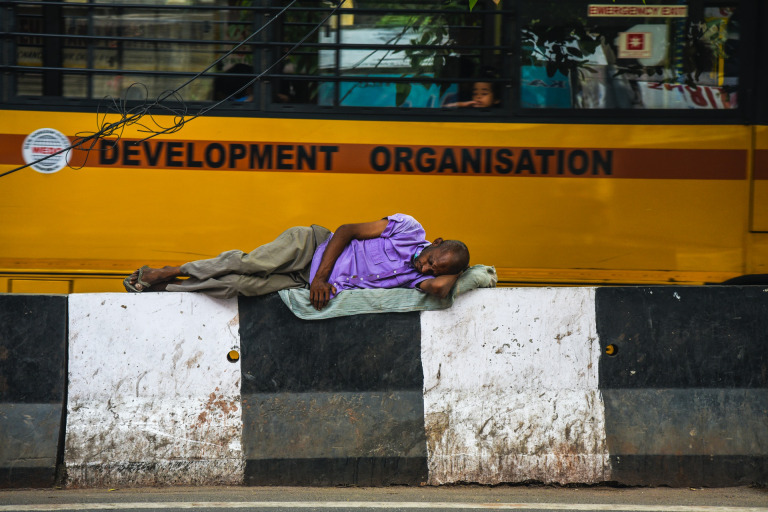
Photo: By Abhishek Goel, freely downloadable from Pexels.com.

Since poorer economies have limited financial resources and weaker health systems, with insufficient health workforce and testing capacity, leaders are acting quickly to try to save lives. Yet, the economic consequences of these measures might devastate their economies even further. Take India for example, where still 60% of the population lives below the World Bank’s median poverty line. The shutdown order sparked mass migration of workers who were forced to walk hundreds of miles to their home villages after public transport was suspended. In Kenya, where President Kenyatta ordered milder measures to slow the coronavirus outbreak, informal laborers, who account for more than 80% percent of the total workforce, are left without jobs. More generally, in developing countries most of the communities do not have access to clean water, let alone soap. Additionally, as individuals mostly rely on their daily income to put food on the table, they are facing the hard choice to either go to work with a chance of getting the virus or assured starvation if they stay at home. There is a concrete risk that people will die of hunger before the curve will flatten [4].

Among the consequences of the coronavirus outbreak in developing economies, something else to consider is how the epidemic could ramp up inequality. First, poorer countries are likely to be hit harder than rich countries, due to poor infrastructure and lack of resources which will hamper public health efforts. This will widen inequality between the developed and developing world. Second, both in the developed and developing world, inequality in the societies are expected to widen as well. Poorer workers relying on daily income will be the most affected as they are at a greater risk of losing their jobs. Another inequality that is expected to increase relates to gender. As 70% of women are health workers and women do more than three-quarters of all unpaid care work [5], women will be the more affected by the crisis compared to men [6]. Finally, some measures like social distancing as well as the availability of clean water remain impossible for people in conflict zones and refugee camps or migrants. Coronavirus could further widen inequality among these vulnerable populations [7].

After the Ebola outbreak in West Africa, one of biggest epidemics to occur in recent history, the international community was criticized for its failure to warn the world in time and for the slow and inadequate response [8]. The World Bank set up a Pandemic Emergency Financing Facility (PEF) – a financing mechanism designed to quickly release funds to the world’s poorest countries and agencies to mitigate the humanitarian and economic consequences of cross-border, large-scale outbreaks. The PEF helped release funding for the 2018-2020 DRC Ebola outbreak, but its design is currently highly criticized for the complex payout criteria which does not allow the release of funding in response to the coronavirus outbreak. Only two outbreaks since 2006 would have met the criteria to activate PEF’s insurance payments, with PEF remaining inflexible or too slow to meet its aim of preventing disease outbreaks from becoming pandemics [9].

As a result, international agencies are now considering acting for developing countries in other ways. The World Bank Group just approved a US$ 1.9 billion fast-track package to strengthen the COVID-19 response in the developing world and shorten the time to recovery [10]. Economists and global health experts have called on G20 leaders to provide financial support to poorer countries for their health systems and economies. Others believe that relieving poorest countries from debt will help them better manage health and economic impacts of the coronavirus. As the developed countries are focused on getting stimulus packages approved for their economy, some poorer countries are thinking of doing the same. India’s finance ministry just announced a US$22 billion economic stimulus package that will include delivering grains and lentil rations for three months to 60 percent of the population. Still, not all developing countries will have the resources to do that.

There is still an urgent need for action to help mitigating the impact of the coronavirus epidemic in less developed countries. As those economies with few recorded cases are already feeling the consequences of the contagion, this is a call to coordinated efforts at the international level. The response should reach those most vulnerable not only to mitigate the health and economic costs of the virus, but more importantly to limit the widening of inequality worldwide.
